# Association between periodontitis and periodontal indices in newly diagnosed bronchial asthma

**DOI:** 10.34172/japid.2022.010

**Published:** 2022-08-06

**Authors:** Amir Moeintaghavi, Afsaneh Akbari, Fariba Rezaeetalab

**Affiliations:** ^1^Dental Research Center, Mashhad University of Medical Sciences, Mashhad, Iran; ^2^Department of Periodontics, School of Dentistry, Mashhad University of Medical Sciences, Mashhad, Iran; ^3^Lung Diseases Research Center, Mashhad University of Medical Sciences, Mashhad, Iran

**Keywords:** Asthma, AST, inflammation, periodontal disease, periodontitis

## Abstract

**Background.** Periodontitis is an inflammatory disease of the tooth-supporting structures. Current data suggest that periodontal disease may be a risk factor for asthma. The present study aimed to assess the prevalence of periodontitis and its relationship with the severity of asthma in asthmatic patients.

**Methods.** This study was conducted on 70 newly diagnosed asthmatic patients as the case group and 70 healthy subjects as the control group, aged 20‒50. The asthma was diagnosed by a pulmonologist according to Global Initiative for Asthma (GINA) guideline. All the participants underwent peri­odontal examinations, which included measuring the pocket depth (PD), attachment loss (AL), gingi­val index (GI), and plaque index (PI) in one tooth from each sextant, including the incisor/canine and left and right premolar/molar regions for both the maxillary and mandibular dental arches.

**Results.** Periodontal disease was significantly more prevalent in newly diagnosed asthma patients. Patients with asthma had significantly higher PI, GI, PD, and AL scores (P<0.001). Furthermore, dry mouth in asthmatic patients with cough and mucosal changes in asthmatic patients with wheeze were significantly more common than in non-asthmatic patients (P<0.05). The median AL in wheezing patients and the median AL and PD in participants who had asthma attacks within the previous month were significantly higher than in other patients. Furthermore, there was a significant negative correlation between AL with Forced expiratory volume in one second (FEV1) and forced vital capacity (FVC) and PD with FEV1 and FVC.

**Conclusion.** Our results showed that periodontal diseases were more prevalent in newly diagnosed asthmatic patients, and asthma was more severe in periodontitis patients.

## Introduction

 Bronchial asthma is a chronic inflammatory disease of the airways that affects people of all ages around the world.^1^ According to the World Health Organization, asthma is a costly chronic disease, affecting around 262 million people globally in 2019 and resulting in 461000 deaths.^2^ Histopathological modifications that cause asthmatic airway malfunction include airway inflammation, subepithelial fibrosis, smooth muscle and goblet cell hyperplasia, hypertrophy, neovascularization, and reduced airway lumen size.^3,4^ Evidence suggests that bronchial asthma, as an inflammatory disease, is influenced by various inflammatory factors such as interleukins and immune cells.^5^ Therefore, inflammation and infection can be risk factors for bronchial asthma and asthma exacerbation.^6,7^ As a result, chronic inflammatory diseases appear to be risk factors for bronchial asthma by activating the immune system and inducing an inflammatory response.

 Current data suggest that periodontal disease may be a risk factor for asthma, emphasizing the correlation between oral hygiene and general systemic health.^8^ For example, previous studies reveal that periodontitis as a disease is associated with a higher risk of atherosclerosis, myocardial infarction, respiratory disease, diabetes, and preeclampsia.^9^ Periodontitis is a chronic inflammation of the periodontium (tooth-supporting tissues) caused by a bacterial infection.^10^ Bacterial biofilm on the teeth, genetics, environmental factors, and some inappropriate behavioral habits are involved in the development and progression of this disease.^11^ In addition, bronchodilators and inhaled medicines used by asthmatic patients are other risk factors for oral hygiene.^12^

 In this regard, some studies have found higher microbial plaque deposition, gingivitis, periodontitis, and reduced salivary flow in asthmatic patients compared to healthy individuals. For example, Kelly et al^13^found a possible association between asthma and periodontal disease in adults after comparing periodontal disease indicators such as plaque index (PI), gingival index (GI), calculus index (CAI), pocket depth (PD), attachment loss (AL), and bleeding on probing (BOP) between asthmatics and healthy individuals. However, they suggested further research to determine the interrelationship of these disorders. Although the exact association between asthma and periodontal disease is unclear, it may involve either pathological activation of the immune and inflammatory responses or side effects of anti-asthma medications, which need to be further considered.^13^ On the other hand, some results are contradictory due to differences in sample size, sociodemographic factors, and diagnostic criteria used to diagnose periodontal disease.

 Therefore, this study aimed to investigate periodontal indices in Iranian asthmatic patients and determine the relationship between these indices and disease severity. The novelty of the research is periodontitis in newly asthmatic patients without inhalation of bronchodilator spray, such as inhaled corticosteroids that affect the orodental system.

## Methods

###  Ethics statement 

 This experimental study was conducted under the Helsinki Declaration to treat human subjects. The study protocol was also approved by the Ethics Committee of Mashhad University of Medical Sciences, Mashhad, Iran, under the code IR.MUMS.DENTISTRY.REC.1399.113. The participants were informed about the research objectives and written informed consent was obtained. Furthermore, participants were guaranteed that their information would be kept confidential, the research protocol would not interfere with their asthma care, and they would not be charged.

###  Sampling

 This control‒case study was conducted on 70 patients with bronchial asthma, who were admitted to the Lung Diseases Research Center, Mashhad University of Medical Sciences, Mashhad, Iran, between 2020 and 2021. The participants were enrolled by convenience sampling. The sample size was calculated using Epi-info software, based on a 40% prevalence of the periodontal disease among asthmatic patients as indicated by Khassawneh et al.^14^ The inclusion criteria were: newly diagnosed with bronchial asthma, an age range of 20‒50, having natural teeth, and the ability to give informed consent. The exclusion criteria for patients were: wearing partial or complete dentures, nonsteroidal anti-inflammatory drugs (NSAIDs) use in the past three months, smoking and opium addiction, history of diabetes mellitus, collagen vascular diseases, rheumatic diseases, epilepsy and seizures, cardiovascular diseases, hypertension, thyroid disease, hyperlipidemia, chronic renal failure, liver cirrhosis, hematologic disorders, malignancy, and endocrine diseases. The control group included healthy volunteers with no history of bronchial asthma, natural teeth, and an age range of 20‒50. At the beginning of the test, the participants’ demographic data were recorded in the sociodemographic characteristic form.

###  Spirometry

 Asthma was diagnosed by a pulmonologist based on spirometry results according to the Global Infinitive for Asthma (GINA) guidelines.^15^ The participants were subjected to a spirometry test using a Micro Medical Spirometer (Micro Medical Ltd., UK) to determine their forced expiratory volume in one second (FEV1). The FEV1 values >80%, 80‒60%, and <60% were considered mild, moderate, and severe, respectively. In addition, forced vital capacity (FVC) and FEV1/FVC were calculated for patients.

###  Asthma control test (ACT) questionnair

 The ACT is a 5-item, self-administered questionnaire used to assess asthma control, independent of pulmonary function measurements. For asthma control, the ACT questionnaire items (14) are rated on a five-point Likert scale from 1 (always) to 5 (never), as well as 1 (not controlled at all) to 5 (completely controlled). The ACT scores vary from 5 (poorly managed asthma) to 25 (well-controlled asthma), with a score of 19 or above suggesting well-controlled asthma. Furthermore, the incidence of asthma symptoms, including dyspnea, coughing, wheezing, chest tightness, pain, the number of attacks per month, and the amount of medication used, were recorded. Spirometry and ACT questionnaires were performed by one experienced pulmonologist (FR).

###  Periodontal examination

 Patients were subjected to a clinical periodontal examination, including measuring the community periodontal index for treatment needs (CPITN), PD, AL, and GI. A dentist examined one tooth (CPITN index teeth) from each sextant, including the incisor/canine and left and right premolar/molar regions for both the maxillary and mandibular dental arches. CPITN was measured using a standardized protocol to investigate gingival bleeding, calculus, and periodontal pockets. The following ten teeth: upper right: 17 and 16; upper anterior: 11; upper left: 26 and 27; lower right: 47 and 46; lower anterior: 31; and lower left: 36 and 37, were examined, and each tooth was given its highest CPITN score ranging from 0 to 4.^16^ All measurements were made in four areas for each tooth (mesiobuccal, mid-buccal, distobuccal, midpalatal/lingual) using a Williams probe (Hu-Friedy, Chicago, IL, USA) according to the standard method. The probe was placed in the periodontal pocket parallel to the longitudinal axis of the tooth; therefore, it did not pass through the junctional epithelium. Plaque index and gingival index (Silness and Loe)^17^ were measured, and the means of the recorded values were considered as the plaque index and gingival index and registered for each subject. Furthermore, dry mouth and decreased saliva, changes in the oral mucosa, the presence of oral candidiasis, gingivitis, oral hygiene status, dental caries clinically (by observing caries cavity, a gray shade beyond the enamel, sticking of the examination probe), and the total number of teeth were examined. Periodontal examinations were carried out by a skilled dentist (AA). They were qualified, calibrated, and approved by a reliable senior professor of periodontology (AM).

###  Statistical analysis

 Statistical analyses were performed by SPSS 21.0 (SPSS Inc., USA). Following the Shapiro–Wilk test to determine the normality of the data, intergroup comparisons were performed using the Student’s t-test for parametric data and the Mann–Whitney U test for nonparametric data. The chi-squared test was also used to compare qualitative variables. The quantitative data were expressed as median (interquartile range) and qualitative data as a percentage. A P-value of <0.05 was considered statistically significant.

## Results

###  Demographic data

 The periodontal indices of 70 asthmatic patients were evaluated in this study to determine the relationship between periodontitis and asthma. The mean ± SD ages of asthmatic patients and healthy controls were 37.67±9 and 38.37±9.7 years, respectively. Furthermore, the asthmatic group consisted of 32 (45.7%) males and 38 (54.3%) females, while the control group included 34 (48.6%) males and 36 (51.4%) females. However, no statistically significant difference in gender distribution was found between asthmatic patients and healthy controls by chi-squared analysis. Furthermore, none of the participants smoked or were drug addicts (Table 1).

**Table 1:** Demographic characteristics in the two groups and clinical presentation in asthmatic patients

**Variables**	**Healthy group (n=70) **	**Asthmatic group (n=70) **	**P-value **
**Age **	38.37±9.7	37.67±9	544 **.0
**Gender (%, N) ** **Female ** **Male **	36 (51.4) 34 (48.6)	38 (54.3) 32 (45.7)	735**.0
**Cough (%, N) **	-	50 (71.4)	-
**Dyspnea (%, N) **	-	64 (91.4)	-
**Wheezing (%, N) **	-	35 (50)	-
**Nocturnal asthma (%, N) **	-	28 (40)	-
**Chest tightness (N, %) **	-	30 (42.9)	-
**Asthma attack (N, %) ** **0** **1** **2** **3 ** **4 **	-	55 (78.6) 5 (7.1) 1 (1.4) 1 (1.4) 8 (11.4)	-
**Emergency visits in the past one month (N, %) **	-	3 (4.3)	-
**FEV1/FVC% (Mean ± SD) ^a^**	-	70%±14.88%	-
**(Mean ± SD) ^b^ FEV1 L/s **	-	2.21±1.30	-
**(Mean ± SD) ^c^ FVC L/s **	-	2.9±1.1	-

N = Number ^a^ FEV1/FVC% = Forced Expiratory Volume/Forced Vital Capacity ^b^ Forced Expiratory Volume /L ^c^ Forced Vital Capacity/ L

###  Clinical features of asthmatic patients

 The ACT questionnaire was employed to assess asthma severity. According to the results, asthma was well controlled in 2 (2.9%) patients, partially in 28 (40%) patients, and uncontrolled in 40 (57.1%) patients. Three (4.3%) of the patients had also experienced an emergency in the last month. Furthermore, coughing, wheezing, dyspnea, and chest tightness were also reported in 50 (71.4%), 35 (50%), 64 (91%), and 30 (42.9%) patients, respectively. Twenty-eight (40%) patients had a history of nocturnal asthma attacks. In addition, 55 patients had no asthma attacks in the previous month; five had one, one had two, one had three, and eight had four attacks. On the other hand, spirometry values revealed that the mean ± SD of FEV1, FVC, and FEV1/FVC ratio were 2.21±1.30 L/s, 2.9±1.1 L/s 70%±14.88%, respectively. According to the results, FEV1/FVC was abnormal in 48 (86.6%) patients.

###  Periodontal parameters

Table 1 compares periodontal parameters and periodontitis severity in asthmatic patients and controls. As demonstrated, there was no statistically significant difference in the number of teeth between the two groups. However, healthy controls had significantly more decayed teeth (P<0.01) and a higher decayed tooth/total-tooth ratio (P<0.05) than patients. In contrast, asthmatics had significantly higher median scores for PI, GI, AL, and PD parameters than controls (P<0.05, Table 2). On the other hand, the CPITN score distributions among groups varied widely. As shown in Table 1, the percentage of people in the control group with CPITN scores of zero and three was significantly higher than that of asthmatic patients (P<0.001).

**Table 2:** Comparison of periodontal indices and tooth decay in newly diagnosed asthmatic patients and healthy controls

**Parameter**		**Patients (n = 70)**	**Controls (n = 70)**	**P-value**
**Total Teeth Number (median)**		26 (8)	27 (5)	0.570 *
**Number of Decayed Teeth (median)**		3 (3)	4 (4)	0.009 *
**ratio of decayed teeth (median)**		0.11 (0.13)	0.15 (0.18)	0.038 *
**Plaque Index (mean ± sd)**		1.3±0.4	1±0.4	< 0.001 **
**Gingival Index (median)**		1.7 (0.8)	1 (0.8)	< 0.001 *
**Attachment Loss (median)**		2.6 (1.7)	1.4 (0.7)	< 0.001 *
**Pocket Depth (median)**		1.7 (0.6)	1.2 (0.7)	< 0.001 *
**^a^Cpitn Score**	0	23 (5.5)	38 (9)	< 0.001 ***
	1	175 (41.7)	153 (36.4)	
	2	185 (44)	148 (35.2)	
	3	27 (6.4)	77 (18.3)	
	4	10 (2.3)	4 (1)	
**Periodontal Status According to Cpitn**	Normal	3 (4.3)	5 (7.1)	0.056 ***
	Gingivitis	48 (68.6)	34 (48.6)	
	Periodontitis	19 (27.1)	31 (44.3)	
**Oral Health Status**	Bad	33 (47.1)	3 (4.3)	< 0.001 ***
	Moderate	27 (38.6)	42 (60)	
	Good and very good	10 (14.3)	25 (35.7)	
**Gingivitis (%)**		67 (95.7)	65 (92.9)	0.466 ***
**Candidiasis (%)**		0	0	-
**Dry Mouth (%)**		26 (37.1)	13 (18.6)	0.014 ***
**Mucosal Changes (%)**		9 (12.9)	4 (5.7)	0.145 ***

*Mann-Whiney test, **Student's t-test, ***chi-squared test ^a^CPITN: Community Periodontal Index of Treatment Needs Oral health status was graded according to the plaque index (PI)12: good and very good: 0-1 rate: 1-1.9 bad: 2-3

 In contrast, the percentage of people in the asthmatic patients’ group with CPITN scores of one, two, and four was significantly higher (P<0.001). Furthermore, most participants had gingivitis, and the groups did not differ significantly in this regard. In addition, candidiasis was also not observed in any of the participants. It should be noted that asthmatic patients had a significantly higher incidence of dry mouth than healthy subjects (P=0.014), and their oral hygiene was considerably worse (Table 2). According to the results, 47% of asthmatic patients had poor oral hygiene compared to 4.3% in the control group, a statistically significant difference (P<0.001).

 Furthermore, we investigated the periodontal indices and oral hygiene in asthmatic patients based on gender. The results revealed that males had significantly weaker oral hygiene status than females, with 62.5% having poor oral hygiene compared to 34.2% (P<0.05). Moreover, 21.9% of males had moderate oral hygiene, and 15.6% had good or excellent oral health. These values were 52.6% and 13.2% in female patients, respectively, which was significantly higher than in males (P<0.05). However, there was no statistically significant difference between male and female patients regarding other parameters.

###  Asthmatic variables

 Based on asthmatic variables, we evaluated the periodontal indices and oral hygiene in asthmatic patients. The results of a chi-squared analysis showed that dry mouth was significantly more common in asthmatic patients with cough (46%) than in those without cough (15%) (P<0.05). Furthermore, the total number of teeth in asthmatic patients who did not have wheezing was 28, which was significantly higher than the 24 teeth in asthmatic patients with wheezing (P=0.008). In addition, the AL and mucosal changes were significantly higher in asthmatic patients with wheeze (P<0.05). Moreover, in patients who had not experienced an asthma attack in the previous month, AL and PD parameters increased from 2.5 and 1.7 to 2.9 and 1.8 in patients who had an asthma attack in the previous month, which was significant (P=0.017, P=0.012).

 On the other hand, dyspnea, chest tightness, nocturnal asthma, and asthma control had no significant effect on periodontal parameters. In addition, correlation analysis of respiratory variables and periodontal indices revealed a significant negative correlation between AL and FEV1 and FVC (Table 3). There was also a significant negative correlation between PD and FEV1 and FVC. However, other respiratory parameters and periodontal indices had no significant relationship (Table 3). Similarly, the correlation between CPITN and FEV1 was not significant (Table 4).

**Table 3:** The correlation coefficient between respiratory variables and periodontal indices

**Parameters**	**FEV1**	**FVC**	**FEV1/FVC**	**ACT**
**PI^a^**	r = -0.034 p = 0.780	r = -0.051 p = 0.520	r = -0.204 p = 0.741	r = -0.028 p = 0.820
**GI^b^**	r = -0.117 p = 0.339	r = -0.079 p = 0.520	r = -0.204 p = 0.093	r = 0.070 p = 0.567
**AL^c^**	r = -0.284 p = 0.017	r = -0.270 p = 0.024=p	r = -0.125 p = 0.302	r = 0.117 p = 0.143
**PD^d^**	r = 0.275 p = 0.021	r = -0.294 p = 0.013	r = -0.083 p = 0.496	r = 0.008 p = 0.947

^a^PI = plaque index ^b^GI = gingival index ^c^AL = attachment loss ^d^PD = pocket depth

**Table 4:** The correlation coefficient between CPITN and FEV1

**FEV1** **CPITN**	**Normal**	**Moderate**	**Severe**	**P-value**
**Normal**	2 (7.7)	0 (0)	1 (3.3)	
**Gingivitis**	17 (65.4)	11 (78.6)	20 (66.7)	0.751*
**Periodontitis**	7 (26.9)	3 (21.4)	9 (30)	
**Total**	26 (100)	4 (100)	30 (100)	

*chi-squared test

## Discussion

 The present study aimed to assess the incidence of periodontitis in asthmatic patients. This research investigated periodontal indicators in asthmatic patients and their relationship with asthma severity. The importance of inflammation and infection as risk factors for bronchial asthma has been considered. Asthma appears to be associated with immune cells and inflammatory mediators like cytokines, leukotrienes, and immunoglobulins. In this regard, periodontal diseases are inflammatory conditions of the oral cavity that cause inflammation of the tissues surrounding the teeth and chronic immune system disturbance. Periodontal diseases aggravate inflammatory and immunological responses by transferring bacteria into the bloodstream, which affects the progression of the disease in patients with bronchial asthma by altering the airway epithelium.

 The present study found a significant increase in periodontal disease in newly diagnosed asthma patients. Despite having a higher number of decayed teeth and higher CPITN scores in healthy controls (P<0.001), asthmatic patients had significantly higher PI, GI, PD, and AL scores (P<0.001). In addition, dry mouth in asthmatic patients with cough and mucosal changes in asthmatic patients with wheeze was significantly more common than in other patients who did not have these symptoms (P<0.05). The median AL in patients with wheeze, and the median AL and PD in participants who had asthma attacks in the previous month, were significantly higher than in other patients.

 Generally, the findings of this study are consistent with previous research on the association between asthma and periodontal disease.^8^ Harshita et al^18^ reported that periodontal diseases are more common in asthmatic patients compared with healthy people. Furthermore, the oral hygiene index in healthy individuals was better than in asthmatic patients, consistent with the current study. They found that the type of asthma medication used by asthmatics affected the extent of periodontitis rather than the severity of asthma. Their findings also showed that, despite the lack of differences in GI between asthmatics and healthy individuals, the mean PD and AL were significantly higher in asthma patients, which was consistent with our findings.^18^ It should be emphasized that measures for AL and PD in the current study were taken between 6 teeth in the CPITN index rather than all teeth. Another study by Shen et al^19^showed that the incidence of periodontal disease was significantly higher in patients with newly diagnosed asthma than in healthy subjects. They suggested that asthmatic patients are more likely to develop periodontal disease.^19^ Furthermore, Yaghoobi et al^20^ reported that periodontal parameters in asthmatic patients were weaker than in healthy subjects, providing additional support to previous studies that advocated an association between respiratory disease and periodontal health status.^20^ In a study on a representative sample of Korean adults, Lee et al^21^ discovered a relationship between asthma and periodontitis in patients with a recent asthma diagnosis. Patients taking anti-asthmatic drugs regularly were also less likely to be diagnosed with periodontitis. According to their findings, poorer oral hygiene was associated with older age, female gender, less physical activity, lower socioeconomic status, smoking, and alcohol consumption.^21^ In a similar study, Moeintaghavi et al^22^found a strong negative correlation between FEV1 and FVC with PI and AL levels in patients with COPD. There was also a significant positive association between the COPD severity score with PI and AL. Similarly, FEV1/FVC was also found to have a negative correlation with PD and AL.^22^ To explain the difference between Moeintaghavi’s work and the present study, it can be concluded that COPD usually has a chronic course and is associated with older age groups who have a history of smoking. Also, evidence indicated that the prevalence of periodontitis in patients with severe asthma was much greater than in the healthy control group. Recent research has suggested that periodontal involvement in asthma may be due to immunological and inflammatory processes, asthma drug adverse effects, or both. The main cause of periodontal disorders is likely a decrease in the protective impact of saliva during the dry mouth. Mouth breathing, changes in salivary composition, or decreased salivary flow can all contribute to this issue. A dry mouth can diminish the concentration of IgA in saliva and disrupt the balance of bacterial and immunological components in the oral cavity. Higher levels of calcium and phosphate in saliva and increased IgE concentrations in gingival tissues can contribute to periodontal diseases.^23,24^ Inhaled corticosteroids used to treat asthma, on the other hand, may lower bone mineral density, including the mandible, and increase the risk of pathological fractures, especially in those using moderate to high doses.^25,26^ Alveolar mucosal dehydration, altered immune response with increased IgE and IgA concentrations, decreased salivary flow, increased mass deposition due to increased salivary calcium and phosphorus levels, and reduced jaw bone density as a result of steroid use are all possible causes of periodontal disease in asthma patients.^12,27^

## Conclusions

 Patients with newly diagnosed asthma are far more likely than healthy individuals to develop periodontal diseases and poor dental hygiene. This study also revealed that periodontal disease is more common in individuals with severe asthma. These findings emphasize the significance of regular oral health monitoring in asthmatic patients, specifically those with severe asthma.

## Limitations

 Due to the impossibility of obtaining dental radiographs for all patients, dental caries was evaluated only clinically (observation of carious cavity, a gray shadow beyond the enamel, and sticking of the examination probe). Therefore, we may not be able to accurately evaluate the results of caries prevalence (especially interdental caries). This may be the reason for the discrepancy between the results of caries prevalence and oral health status in the two groups of asthmatic and healthy subjects.

## Acknowledgments

 This study was financially supported by the Research Vice-Chancellor, Mashhad University of Medical Sciences (Ethical code: IRMUMSDENTISTRYREC.1399.113).

## Competing Interests

 The authors declare no conflict of interest related to the study.

## Authors’ Contributions

 AM, AA, and FR designed and carried out the research, collected the data, interviewed the patients, coordinated the study, participated in the research and prepared the manuscript. AA performed the statistical analyses and revised the English manuscript. All authors have read and approved the content of the manuscript.

## Funding

 There was no funding support for the study.

## Availability of data

 The datasets used and/or analyzed during the current study are available from the corresponding author on reasonable request.

## Ethics Approval

 (Ethical code: IRMUMSDENTISTRYREC.1399.113).
